# Cytotoxicity of Oleandrin Is Mediated by Calcium Influx and by Increased Manganese Uptake in *Saccharomyces cerevisiae* Cells

**DOI:** 10.3390/molecules25184259

**Published:** 2020-09-17

**Authors:** Lavinia L. Ruta, Claudia V. Popa, Ileana C. Farcasanu

**Affiliations:** Department of Organic Chemistry, Biochemistry and Catalysis, Faculty of Chemistry, University of Bucharest, Sos. Panduri 90-92, 050663 Bucharest, Romania; lavinia.ruta@chimie.unibuc.ro (L.L.R.); valentina.popa@chimie.unibuc.ro (C.V.P.)

**Keywords:** oleandrin, *Saccharomyces cerevisiae*, calcium, manganese

## Abstract

Oleandrin, the main component of *Nerium oleander* L. extracts, is a cardiotoxic glycoside with multiple pharmacological implications, having potential anti-tumoral and antiviral characteristics. Although it is accepted that the main mechanism of oleandrin action is the inhibition of Na^+^/K^+^-ATPases and subsequent increase in cell calcium, many aspects which determine oleandrin cytotoxicity remain elusive. In this study, we used the model *Saccharomyces cerevisiae* to unravel new elements accounting for oleandrin toxicity. Using cells expressing the Ca^2+^-sensitive photoprotein aequorin, we found that oleandrin exposure resulted in Ca^2+^ influx into the cytosol and that failing to pump Ca^2+^ from the cytosol to the vacuole increased oleandrin toxicity. We also found that oleandrin exposure induced Mn^2+^ accumulation by yeast cells via the plasma membrane Smf1 and that mutants with defects in Mn^2+^ homeostasis are oleandrin-hypersensitive. Our data suggest that combining oleandrin with agents which alter Ca^2+^ or Mn^2+^ uptake may be a way of controlling oleandrin toxicity.

## 1. Introduction

**Figure 1:**
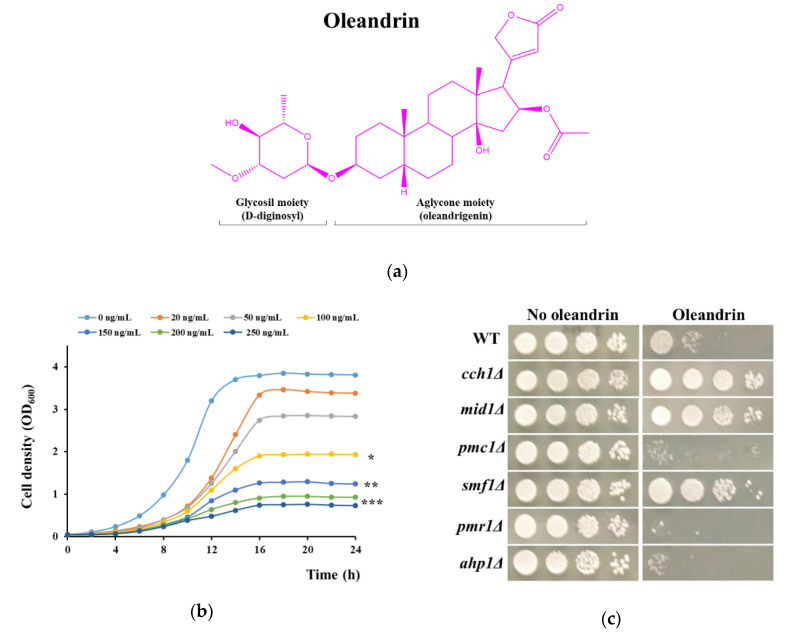
Effect of oleandrin on yeast growth. (**a**) Oleandrin structure. (**b**) Effect of oleandrin concentration on growth of wild type cells. BY4741 cells were inoculated (OD_600_ = 0.05) and grown in SD (synthetic dextrose) liquid medium in the presence of various concentrations of oleandrin. Cell density was determined spectrophotometrically at 600 nm (OD_600_) as described in the *Materials and Methods* section. One-way ANOVA, * *p* < 0.05; ** *p* < 0.01; *** *p* < 0.005. (**c**) Growth on oleandrin-supplemented solid medium. Wild type (WT) or knockout mutant cells *cch1Δ*, *mid1Δ*, *pmc1Δ*, *smf1Δ*, *pmr1Δ* and *ahp1Δ* with oleandrin sensitivity different from WT (see Table S1) were serially diluted and stamped on SD/agar containing or not 100 ng/mL oleandrin. Plates were photographed after 3 days’ incubation at 30 °C.

*Nerium oleander* L., commonly known as oleander, is an ornamental shrub with both pharmacological and toxicological properties, whose parts have been used in ethnomedicine since ancient times as natural remedies against cardiac illnesses, cancer, diabetes, asthma, skin diseases, inflammation, etc. [1]. Oleander extracts need to be regarded with caution as they are poisonous in high doses, having important cardiotoxic effects [2,3,4]. Nevertheless, many of the individual components of oleander extracts have been found to have anti-tumor, anti-proliferative, anti-inflammatory and even antiviral properties [5,6,7,8]. One of the emblematic components of oleander extracts is oleandrin (PubChem CID 11541511), a cardiotonic glycoside similar in toxicity and structure to digitoxin from *Digitalis purpurea* L. [7]. Oleandrin (Figure 1a) is a lipid-soluble glycoside comprised of oleandrigenin (the steroid aglycone) and D-diginosyl (a sugar-like moiety) which slightly increases oleandrin’s water solubility, which otherwise is very low [9]. Oleandrin is mainly responsible for the toxicity of oleander sap and, just as with digitoxin, it is thought to act as a cardiotonic by inhibiting the sodium and potassium ATPases (Na^+^/K^+^-ATPase) and subsequently increasing Ca^2+^ concentration, resulting in activation of various cell survival and death pathways [10,11]. In spite of its toxicity, oleandrin has been increasingly investigated as several studies indicated its potential as an anticancer [12,13,14,15,16] as well as antiviral drug [8,17,18,19]. The therapeutic potential of oleandrin is hampered by its cytotoxicity and by the fact that although several signaling cascades targeted by oleandrin through inhibition of Na^+^/K^+^-ATPase have been identified [20], and that it is considered that oleandrin may cause destruction of tumor cells by inducing oxidative stress through generation or reactive oxygen species (ROS) [21], many aspects which mediate oleandrin toxicity still remain obscure.

In this study, we made use of the model microorganism *Saccharomyces cerevisiae* to investigate oleandrin toxicity on yeast cells. *S. cerevisiae* is a simplified model of the eukaryotic cell used to elucidate many of the molecular mechanisms conserved in higher eukaryotes due to the ease of manipulation, tractable genetics, exhaustive genome annotation and less restrictive ethical constraints [22,23,24]. So far, no study concerning the effect of oleander extracts or oleandrin has been reported in *S. cerevisiae*. As oleandrin was shown to alter the fluidity of the human cell membrane [25], we hypothesized that the primary interaction between oleandrin and the yeast cells would occur at the plasma membrane level. We therefore tested the oleandrin toxicity on *S. cerevisiae* mutants with defects in the cell membrane transport of monovalent ions (Na^+^ and K^+^), Ca^2+^ or essential metal ions.

In *S. cerevisiae*, the movement of Na^+^ and K^+^ across the plasma membrane is ensured by Ena1 P-type Na^+^/H^+^ ATPase, Nha1 Na^+^/H^+^ antiporter, outward-rectifier K^+^ channel Tok1 and the Trk1p–Trk2p potassium transport system [26]. Regulation of these transporters has been extensively reviewed [26] and it was shown that salt stress and alkaline stress induce calcium-mediated responses by generating Ca^2+^ flux into the cytosol [27,28]. Abrupt increases in the cytosolic Ca^2+^ ([Ca^2+^]_cyt_) represent a universal mechanism to trigger signaling cascades involved in cell adaptation, survival or death [29]. In *S. cerevisiae*, the increase in [Ca^2+^]_cyt_ occurs through Ca^2+^ entry into the cytosol via the Cch1/Mid1 channel situated at the plasma membrane [27,30] or via the vacuolar transient receptor potential channel TRPY1 (formerly known as Yvc1) [31,32,33]. Cch1 is similar to the pore-forming subunit (α1) of the plasma membrane, and voltage-gated Ca^2+^ channels (VGCC) from higher eukaryotes, including humans [34]. Cch1 interacts and partially co-localizes with Mid1p, a stretch-activated cation channel which resembles the VGCC α2/δ regulatory subunits and Na^+^ leak channel non-selective (NALCN)-associated proteins [35]. Prolonged high [Ca^2+^]_cyt_ is detrimental to cells, therefore the normal very low [Ca^2+^]_cyt_ must be restored through the action of Ca^2+^ pumps and exchangers [33]. In *S. cerevisiae*, this is done by the concerted actions of the vacuolar Ca^2+^-ATPase Pmc1 (similar to mammalian PMCA1a) [36] and of the vacuolar Ca^2+^/H^+^ exchanger Vcx1 [37,38] (which independently transport [Ca^2+^]_cyt_ into the vacuole) and by the secretory Ca^2+^-ATPase Pmr1, which pumps [Ca^2+^]_cyt_ into the endoplasmic reticulum (ER) and Golgi [39,40]. Pmr1p is the prototype of a family of transporters known as SPCA (Secretory Pathway Ca^2+^-ATPases) with members found also in mammals [41]. Defects in the human ortholog of PMR1, ATP2C1, are associated with Hailey-Hailey disease [42].

As oleandrin was shown to sensitize human osteosarcoma cells to cisplatin by preventing degradation of the copper transporter CTR1 [15], we also studied the cytotoxicity of oleandrin against *S. cerevisiae* mutants with defects in the transport of essential metal ions across the plasma membrane. In *S. cerevisiae*, there is an intricate system of transporters involved in the high- or low-affinity transport of essential metals, with both high and low specificity [43], e.g., Ctr1 (Cu^+^ transporter [44]), Fet3/Ftr1 (complex involved in the transport of Fe^3+^ and Cu^2+^, [45]), Fet4 (low-affinity transporter for Fe^2+/3+^ and other transition metal ions [46]), Pho84 (phosphate transporter and a low-affinity divalent metal transporter [47]), Smf1 (divalent metal ion transporter with broad specificity and with high affinity for Mn^2+^ [48,49]), Zrt1 (high-affinity Zn^2+^ transporter [50]) and Zrt2 (low-affinity Zn^2+^ transprter [51]). The data obtained in this study indicated that oleandrin exposure induced transient elevations in [Ca^2+^]_cyt_, but also an unexpected increase in Smf1-dependent Mn^2+^ accumulation which was accountable for the increased sensitivity to oleandrin of mutants with defects in Mn^2+^ homeostasis.

## 2. Results

### 2.1. Toxicity of Oleandrin towards Saccharomyces cerevsiae Cells

The investigation of the potential action of oleandrin upon *S. cerevisiae* cells is problematic due to the low solubility of oleandrin in aqueous environments, which does not surpass 1.5 mg/L [9]. We firstly prepared a saturated aqueous solution of oleandrin which was added to yeast culture media at various sub-saturation ratios. It was noted that media containing 500 ng/mL (approximately 1/3 saturation) completely killed the yeast cells, meaning that the toxicity studies would not be hampered by the uneven distribution of oleandrin within the yeast growth media. We further checked the growth of *S. cerevisiae* cells in liquid media supplemented with various concentrations of oleandrin (Figure 1b). It was noted that the presence of oleandrin in the incubation medium affected cell proliferation; the half minimal inhibition concentration calculated after 16 h of incubation was IC_50_ = (99.57 ± 1.25) ng/mL which corresponds to a molar concentration of approximately 0.16 µM.

As the presence of oleandrin in the incubation media affected yeast cell growth, we sought to identify molecular targets of oleandrin toxicity. Considering the oleandrin structure (a glycoside containing a steroid aglycone, Figure 1a), it is highly probable that it primarily interacts with components of the plasma membrane.

Starting from the known facts that in mammalian cells oleandrin: (1) interacts with Na^+^/K^+^-ATPase; (2) induces cell Ca^2+^ elevations; (3) inhibits Cu^+^ transporter Ctr1; and (4) mediates oxidative stress by generation of reactive oxygen species (ROS), we tested the toxicity of oleandrin against yeast mutants hosting individual knockout deletions in genes related to: (1) Na^+^ or K^+^ transport across the plasma membrane; (2) Ca^2+^ transport and homeostasis; (3) heavy metal transporters; and (4) response to oxidative stress. The knockout genes were selected based on a search in the *Saccharomyces* Genome Database (SGD, [52]) and are presented in Table S1. The screening of oleandrin toxicity against the yeast knockout strains was done by exposing cells to an oleandrin concentration which caused approximately half inhibition of wild type growth (i.e., 100 ng/mL). The strains which significantly grew better or worse than the wild type in the presence of oleandrin were selected for further investigation. As shown in Table S1, no mutant with defects in Na^+^ or K^+^ transport showed a different phenotype from the wild type. From the group of mutants with defects in calcium transport, *cch1Δ* and *mid1Δ* grew better, while mutants *pmc1Δ* and *pmr1Δ* were more sensitive to oleandrin than the wild type cells (Table S1, Figure 1c).

From the group of mutants with defects in essential metal transport, only *smf1Δ* cells grew considerably better in the presence of oleandrin (Table S1, Figure 1c). In the case of the mutants defective in the response to oxidative stress, solely *ahp1Δ* grew differently in the presence of oleandrin, being more sensitive than the wild type cells (Table S1, Figure 1c, bottom).

### 2.2. Oleandrin Induces Calcium Influx via Cch1/Mid1

**Figure 2:**
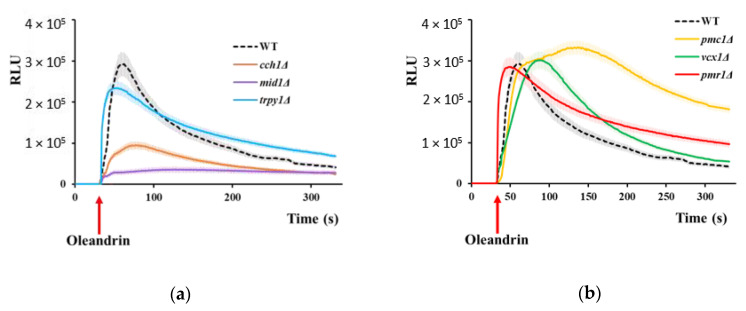
Increase in [Ca^2+^]_cyt_ under oleandrin exposure. Wild type cells or cells defective in calcium transport expressing reconstituted aequorin were pre-grown in SD-Ura and subjected to oleandrin stress (100 ng/mL) as described in *Materials and Methods*. [Ca^2+^]_cyt_-dependent aequorin luminescence was recorded on samples of approximately 10^7^ cells (OD_600_ = 1). The arrow indicates the time when the oleandrin was added. (**a**) Calcium-dependent luminescence of wild type, and of mutants with defects in Ca^2+^ release to the cytosol *cch1Δ*, *mid1Δ and trpy1Δ*. (**b**) Calcium-dependent luminescence of type, and of mutants with defects in Ca^2+^ removal from cytosol *pmc1Δ*, *vcx1Δ* and *pmr1Δ*. The luminescence traces represent the mean ± SEM from 3 independent transformants. SEM are illustrated as bars of a lighter nuance. RLU, relative luminescence units.

The observation that both *cch1Δ* and *mid1Δ* were more tolerant to oleandrin than the wild type cells suggested the idea that oleandrin cytotoxicity is mediated by calcium influx, which in *S. cerevisiae* occurs primarily via the Cch1/Mid1 channel. To test this possibility, we used transgenic yeast cells expressing aequorin, a photoprotein whose luminescence varies as a function of calcium fluctuations; the aequorin-based system used is suitable for detecting transient modifications in [Ca^2+^]_cyt_ [53]. For this purpose, wild type cells, as well as cells with defects in calcium transporters (*cch1Δ*, *mid1Δ*, *pmc1Δ*, *vcx1Δ*, *pmr1Δ* and *trpy1Δ*) were transformed with a plasmid harboring the cDNA of apo-aequorin under the control of a constitutive promoter which afforded an abundant expression of aeqorin in the cytosol [54]. Before oleandrin exposure, the cells expressing apo-aequorin were pre-treated with its cofactor coelenterazine to reconstitute the functional aequorin. The cells expressing functional aequorin were stimulated with oleandrin directly in the luminometer tube. It was noted that the luminescence of wild type cells expressing functional aequorin significantly increased when cells were exposed to half-inhibitory concentrations of oleandrin (100 ng/mL), an indication of the [Ca^2+^]_cyt_ elevation induced by oleandrin shock (Figure 2a).

The onset of [Ca^2+^]_cyt_ elevation coincided with oleandrin addition and it took 30–40 s before luminescence started to decrease, to reach the basal low level after approximately 300 s (Figure 2a, black dashed line). [Ca^2+^]_cyt_ elevation occurs when Ca^2+^ enters the cell from outside via the Cch1/Mid1 channel or is released from the vacuole via the TRPY1 channel (or both). Apparently, the oleandrin-dependent rise in [Ca^2+^]_cyt_ was predominantly of external origin, since *cch1Δ* or *mid1Δ* cells expressing functional aequorin exhibited significantly lower oleandrin-dependent [Ca^2+^]_cyt_ elevation (Figure 2a, brown and purple line, respectively), while *trpy1Δ* cells expressing aequorin showed high oleandrin-induced luminescence (Figure 2a, blue line).

Since *cch1Δ* and *mid1Δ* were more tolerant to oleandrin than the wild type, it can be speculated that [Ca^2+^]_cyt_ elevations mediate oleandrin toxicity. In this line of evidence, the luminescence traces shown by the oleandrin-hypersensitive *pmc1Δ* had a broader pattern compared to wild type, and with no sign of restoring the basal [Ca^2+^]_cyt_ within the 300 s interval characteristic to the wild type (Figure 2b, orange line). This observation suggested that Pmc1 (and not Vcx1, Figure 2b, green line) is crucial for restoring the low levels of [Ca^2+^]_cyt_ following an oleandrin-induced calcium wave by transporting Ca^2+^ to the vacuole and that high Ca^2+^ lingering in the cytosol of *pmc1Δ* cells is responsible for their hypersensitivity to oleandrin. Neither *vcx1Δ* (lacking the vacuolar Ca^2+^/H^+^ exchanger which transports [Ca^2+^]_cyt_ back to the vacuole) nor *trpy1Δ* (lacking the channel which releases Ca^2+^ from the vacuole into the cytosol) showed higher sensitivity to oleandrin than the wild type (Table S1, Figure 1c). Although the luminescence traces of aequorin-expressing *trpy1Δ* and *vcx1Δ* were slightly different, in both cases, [Ca^2+^]_cyt_ decreased significantly after 300 s from oleandrin exposure (Figure 2a, blue line and Figure 2b, green line, respectively). Surprisingly, although *pmr1Δ* expressing functional aequorin showed luminescence traces similar to *trpy1Δ* (Figure 2b, red line, compared to Figure 2a, blue line), *pmr1Δ* cells were hypersensitive to oleandrin (Table S1, Figure 1c).

### 2.3. Oleandrin Exposure Induces Manganese Accumulation

Among the yeast mutants with deletions in the genes encoding essential metal transporters, *smf1Δ* cells manifested increased tolerance to oleandrin. Smf1 is a divalent metal ion transporter with broad metal specificity for divalent and trivalent metals, and with high affinity for manganese [47], therefore we wondered if oleandrin exposure may be accompanied by accumulation of trace metals, eventually Smf1-dependent. To test this possibility, we performed multi-elemental analysis of yeast cells exposed to oleandrin (Table 1). To avoid inherent variation in trace metal composition of the standard media, we grew the cells in a synthetic medium (MMe) with controlled metal concentrations, containing Co^2+^, Cu^2+^, Fe^3+^, Mn^2+^, Ni^2+^ and Zn^2+^ (1 µM each). In this synthetic medium, Li^+^ was also added (final concentration 1 µM) as a replacement for Na^+^. The metal concentrations used were completely non-toxic to cells, even if they were slightly higher than in the standard media (which contain only 0.1 µM Cu^2+^, ultra-traces of Co^2+^ and Ni^2+^ and no Li^+^). It was noticed that, of all the metal ions present in the growth media, only manganese accumulation seemed to be stimulated by oleandrin (Table 1). 

**Figure 3:**
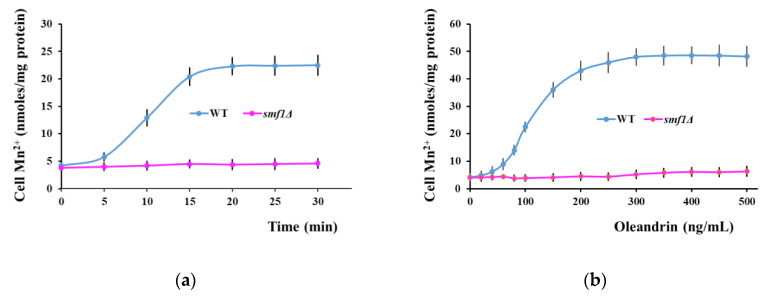
Oleandrin induces Smf1-dependent Mn^2+^ accumulation in yeast cells. (**a**) Time course of Mn^2+^ accumulation following oleandrin exposure. Exponentially growing wild type and *smf1Δ* cells were treated with oleandrin (100 ng/mL) and samples were collected every 5 min for Mn^2+^ assay. (**b**) Effect of oleandrin concentrations on Mn^2+^ accumulation. Exponentially growing wild type and *smf1Δ* cells were treated with increasing oleandrin concentrations and samples were collected after 20 min for Mn^2+^ assay. Cells were incubated (30 °C, 200 rpm) in liquid MMe containing 1 μM MnCl_2_. *** *p* < 0.005, Student’s *t* test.

To check Smf1 involvement, we monitored Mn^2+^ accumulation by wild type and *smf1Δ* cells exposed to 100 ng/mL oleandrin. It was noted that in wild type cells, Mn^2+^ accumulation was induced by oleandrin and increased progressively in the first 5–15 min of exposure; after that, Mn^2+^ accumulation reached a stationary phase (Figure 3a, blue line). No oleandrin-induced Mn^2+^ accumulation could be detected in *smf1Δ* cells, indicating that oleandrin stimulated Mn^2+^ accumulation via the Smf1 transporter (Figure 3a,b purple lines). The Mn^2+^ accumulation was dose-dependent, as Mn^2+^ accumulation increased with oleandrin concentration, to reach a plateau when cells were exposed to oleandrin concentrations higher than 200 ng/mL (Figure 3b, blue line).

### 2.4. Oleandrin Hypersensitivity of Mutants pmr1Δ and ahp1Δ Is Caused by Mn^2+^ Accumulation

**Figure 4:**
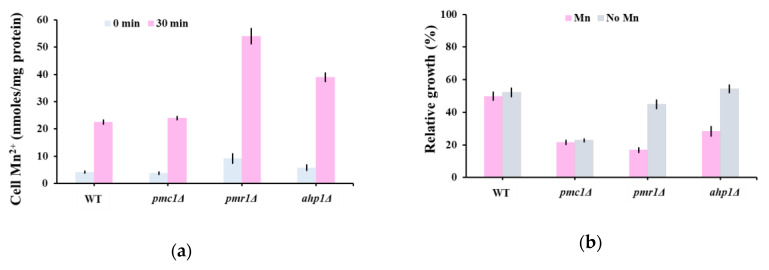
(**a**) Mn^2+^ accumulation by mutants hypersensitive to oleandrin. Exponentially growing yeast cells were treated with oleandrin (100 ng/mL) in MMe containing 1 μM MnCl_2_ and samples were collected after 20 min for Mn^2+^ assay. (**b**) Effect of Mn^2+^ depletion on oleandrin toxicity. Cells were incubated (30 °C, 200 rpm) in liquid MMe containing 1 μM MnCl_2_ (Mn) or Mn^2+^-depleted (No Mn) in the presence of 100 ng/mL oleandrin. Cell growth was measured spectrophotometrically (OD6_00_) and expressed relatively to growth under the same conditions, but in the absence of oleandrin.

The oleandrin hypersensitivity of *pmr1Δ* (Figure 1c, Table S1) could not be explained by the calcium cytosolic wave that followed the oleandrin shock on aequorin expressing *pmr1Δ* (Figure 2b, red line) and had a similar pattern with that of *trpy1Δ* (Figure 2a, blue line), whose oleandrin sensitivity was similar to that of wild type cells (Figure 1c, Table S1). Pmr1 is a high-affinity Ca^2+^/Mn^2+^ P-type ATPase involved in Ca^2+^ and Mn^2+^ transport into Golgi; further, via the secretory pathway, excess Mn^2+^ is extruded from the cell, which is a major route for yeast cells of Mn^2+^ detoxification [40,55]. We therefore wondered if *pmr1Δ* hypersensitivity to oleandrin is related to cells’ incapacity to excrete the excess Mn^2+^ which occurs during oleandrin exposure, rather than to a defect in Ca^2+^ homeostasis. Indeed, it was noted that *pmr1Δ* accumulated significantly more Mn^2+^ than the wild type cells (Figure 4a). Instead, *pmc1Δ* cells which exhibited oleandrin hypersensitivity similarly to *pmr1Δ* displayed Mn^2+^ accumulation which was not significantly different from the wild type (Figure 4a), supporting the idea that *pmc1Δ* oleandrin hypersensitivity is caused by the incapacity of *pmc1Δ* cells to reduce the high [Ca^2+^]_cyt_ levels in due time (Figure 2b).

It was noted that from the group of mutants with defects in the response to antioxidant stress, only *ahp1Δ* exhibited altered oleandrin sensitivity compared to wild type cells. Ahp1 is a thiol-specific peroxiredoxin that reduces hydroperoxides to protect against oxidative damage [56] and that also has a minor role in Mn^2+^ intracellular trafficking [57]. We found that *ahp1Δ* also accumulated more Mn^2+^ than the wild type in the presence of oleandrin (Figure 4a), thus explaining the *ahp1Δ* hypersensitivity. To check that the increased toxicity of oleandrin to *pmr1Δ* and *ahp1Δ* mutants is caused by the increased influx of external Mn^2+^, we determined the relative growth of yeast cells in a Mn^2+^-depleted medium. Indeed, we found that Mn^2+^ depletion improved the growth of *pmr1Δ* and *ahp1Δ* (Figure 4b), indicating that increased Mn^2+^ influx is responsible for the oleandrin hypersensitivity of these mutants. In this line of evidence, the oleandrin sensitivity of *ahp1Δ* was not alleviated by antioxidants known to improve *ahp1Δ* growth, such as ascorbate or tocopherol (data not shown).

## 3. Discussion

**Figure 5:**
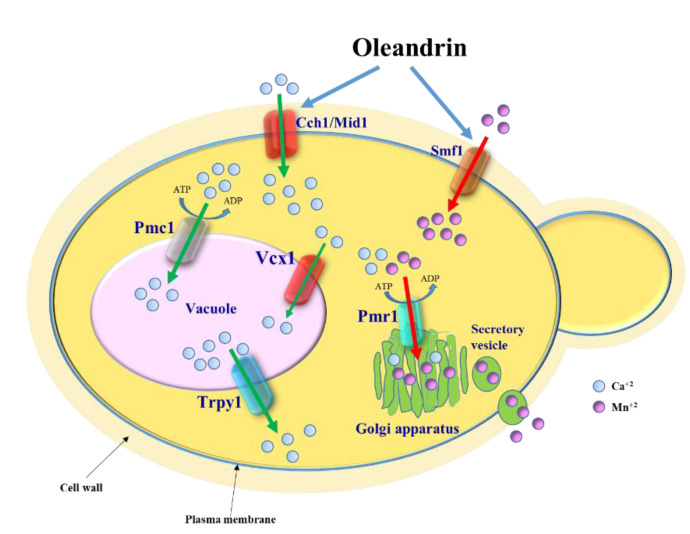
Model depicting the effect of oleandrin on *S. cerevisiae* cells. Oleandrin stimulates Ca^2+^ influx via the Cch1/Mid1 channel and Mn^2+^ uptake via the Smf11 transporter. Excess of [Ca^2+^]_cyt_ is transported into the vacuole via the Pmc1 ATPase pump and Vcx1 transporter. Cells lacking Pmc1 become oleandrin-hypersensitive due to a delay in restoring the low level of [Ca^2+^]_cyt_ (Figure 3b, orange line). The excess of Mn^2+^ taken up via Smf1 in response to oleandrin exposure is removed by the Ca^2+^/Mn^2+^-ATPase pump Pmr1 which transports the excess Mn^2+^ to the Golgi apparatus to be further secreted from the cell via secretory vesicles.

Apart from being a cardiac glycoside, oleandrin has been gathering attention due to its anti-tumoral [12,13,14,15,16] and antiviral potential [8,17,18,19], but its pharmacological use is held back by its variable toxicity. In this study, using the model *S. cerevisiae*, we detected oleandrin-induced fluctuations in cell Ca^2+^, which could be related to the Ca^2+^ entry via the Cch1/Mid1 plasma membrane channel (Figure 5).

At this point, it is hard to determine whether the Ca^2+^ influx was the result of the oleandrin interaction with the membrane transport of monovalent ions, since no yeast mutant with defective Na^+^ or K^+^ transport showed any alteration in oleandrin-mediated toxicity. Although the direct action of oleandrin on the Ena1 Na^+^/K^+^-ATPase cannot be ruled out since *ENA1* is expressed only under salt stress conditions [26], it is highly possible that calcium influx is also triggered by a non-specific interaction of oleandrin with the yeast plasma membrane. In this line of evidence, *cch1Δ* cells, which still have a functional Mid1, showed some calcium influx into the cytosol (Figure 2a). Since Mid1 is a stretch-activated Ca^2^+-permeable cation channel [58], it is possible that oleandrin activates it by mechanical intercalation in the membrane phospholipid layer.

What raised our interest was the unexpected role of oleandrin in stimulating Mn^2+^ accumulation by yeast cells via the transporter Smf1. Mn^2+^ is an essential trace metal that serves as a cofactor for several enzymes [59], which becomes toxic when its concentration surpasses the physiological limits. In yeast, it was shown that divalent metal transport and toxicity can be manipulated by addition of natural compounds such as amino acids or polyphenols [60,61], therefore it would be interesting to screen for synergies between oleandrin and other natural compounds.

It has been suggested that the oleandrin toxicity against certain tumoral cell lines may be the result of ROS generation, especially superoxide ion radicals [21]. Nevertheless, we found that yeast mutants *sod1Δ* (lacking the cytosolic Cu/Zn-superoxide dismutase SOD1) or *sod2Δ* (lacking the mitochondrial Mn-superoxide dismutase SOD2) showed no increased oleandrin sensitivity compared to the wild type cells (Table S1). It is known that supplementary Mn^2+^ can suppress the oxidative damage in yeast cells lacking superoxide dismutase due to the intrinsic superoxide scavenger activity [62], therefore it is possible that in the yeast SOD mutants, the oleandrin-generated superoxide toxicity was counterbalanced by the oleandrin-induced Mn^2+^ influx via Smf1. Either way, it became clear that the effect of oleandrin on the eukaryotic cell may be a multi-facets process, with molecular aspects still waiting to be unraveled.

Oleandrin is of pharmacological interest due to its potential antiviral or anti-tumoral actions; it is tempting to speculate that oleandrin toxicity could be tuned by changing the Mn^2+^ microenvironment of the virus-infected cells or of the tumoral cells. In *S. cerevisiae*, oleandrin stimulated the Smf1-dependent Mn^2+^ influx. Smf1 is a member of the natural resistance-associated macrophage protein (NRAMP) family of transporters which includes the human counterparts DMT1 [63] and NRAMP1 [64] transporters. As NRAMP1 polymorphism has been associated with some types of cancer [65,66], it would be worthwhile studying the oleandrin action on these type of cancer in correlation with Mn^2+^ homeostasis, considering that Mn^2+^ alone was found to inhibit the viability of cancer cells [67,68].

## 4. Materials and Methods

### 4.1. Yeast Strains and Cultivation Media

The *S. cerevisiae* diploid strains used in this study were isogenic with the wild type (WT) parental strain BY4741 (*MAT***a** *his3Δ1*; *leu2Δ0*; *ura3Δ0*), a S288C-based yeast strain [69]. The knockout strains used harbored individual deletions in *YFG* (Your Favourite Gene) and had the genotype (BY4741, *yfg::kanMX4*/*ORF)*, being denoted throughout the manuscript as *yfgΔ*. The strains are presented in Table S1 and they were obtained from EUROSCARF (European *S. cerevisiae* Archive for Functional Analysis). Yeast strains were propagated, grown and maintained in YPD medium (1% *w*/*v* yeast extract, 2% *w*/*v* polypeptone, 2% *w*/*v* glucose) or SD (synthetic dextrose, 0.17% *w*/*v* yeast nitrogen base without amino acids, 0.5% *w*/*v* (NH_4_)_2_SO_4_, 2% *w*/*v* glucose, supplemented with the necessary amino acids) [70]. The strains transformed with the plasmids harboring apoaequorin cDNA [53] were selected and maintained on SD lacking uracil (SD-Ura). For luminescence detection, cells were suspended in SD-Ura supplemented with 2 mM CaCl_2_. Minimal defined media containing known concentrations of metal ions (MMe) were prepared adding individual components as described [70] using ultrapure reagents and contained 1 µM of CoCl_2_; CuCl_2_; FeCl_3_; MnCl_2_; NiCl_2_; ZnCl_2_; and LiCl. The concentrations of metals in MMe were confirmed by inductively coupled plasma mass spectrometry (ICP-MS) (Perkin-Elmer ELAN DRC-e, Concord, ON, Canada). All synthetic media had their pH adjusted to 6.5. For solid media, 2% agar was used. For growth improvement, all the synthetic media were supplemented with an extra 20 mg/L leucine [71]. All chemicals, including media reagents, were from Merck (Darmstadt, Germany). Oleandrin was from Sigma-Aldrich (St. Louis, MO, USA) (Catalog O9640, discontinued) and was of ≥ 98% purity.

### 4.2. Plasmid and Yeast Transformation

For heterologous expression of apo-aequorin, yeast strains were transformed with the multicopy *URA3*-based plasmid pYX212-*cytAEQ* harboring the apoaequorin cDNA under the control of the strong *TPI* yeast promoter [54]. Plasmid pYX212-*cytAEQ* was a generous gift from E. Martegani and R. Tisi (University of Milano-Bicocca, Milan, Italy). Yeast transformation [72] was performed using S.c. EasyComp™ Transformation Kit (Invitrogen, Catalog number: K505001) following the manufacturer’s indications.

### 4.3. Detection of Oleandrin Effect on Yeast Cell Growth

Wild type and *yfgΔ* cells were inoculated from YPD-exponentially growing cells to SD liquid medium (at OD_600_ = 0.05) containing various concentrations of oleandrin added from a 10 mg/mL ethanol stock. Strain growth was monitored in time by measuring the turbidity of cell cultures at 600 nm (OD_600_) recorded in a plate reader equipped with a thermostat and shaker (Varioskan, Thermo Fisher Scientific, Vantaa, Finland). For growth on solid medium, exponentially growing cells (OD_600_ = 0.5) were decimally serially diluted in a multiwell plate and stamped on SD/agar plates containing oleandrin (added after medium sterilization) using a pin replicator (approximately 4 µL/spot). Plates were incubated at 30 °C for 3 days before being photographed.

### 4.4. Detection of [Ca^2+^]_cyt_ by Recording Aequorin Luminescence

Cells transformed with pYX212-*cytAEQ* [54] were maintained on SD-Ura selective medium and prepared for Ca^2+^-dependent luminescence detection as described [73], with slight modifications. Exponentially growing yeast cells expressing apo-aequorin were diluted (OD_600_ = 0.5) in SD-Ura and then incubated to OD_600_ = 1. Cells were concentrated by centrifugation to OD_600_ = 10. To reconstitute functional aequorin, native coelenterazine was added to the cell suspension (from a methanol stock, 20 µM final concentration) and the cells were incubated for 2 h at 30 °C in the dark. Cells were washed to remove the excess coelenterazine and re-suspended in SD-Ura supplemented with 2 mM CaCl_2_. The cells were transferred (approximately 10^7^ cells/determination) to the luminometer tube and a cellular luminescence baseline was determined for each strain by approximately one minute of recordings at 1/s intervals. After ensuring a stable signal, oleandrin was injected (*v*/*v*) from a sterile 200 ng/mL solution in SD-Ura medium, to give the final oleandrin concentration 100 ng/mL (approximately corresponding to half minimal inhibitory concentration, IC_50_). The Ca^2+^-dependent light emission was monitored in a single-tube luminometer (Turner Biosystems, 20^n^/20, Sunnyvale, CA, USA). The light emission was measured at 1 s intervals and expressed as relative luminescence units (RLU). To ensure that the total reconstituted aequorin was not limiting in our assay, at the end of each experiment, aequorin activity was checked by lysing cells with 1% Triton X-100 with 5 mM CaCl_2_ and only the cells with considerable residual luminescence were considered. Relative luminescence emission was normalized to an aequorin content giving a total light emission of 10^6^ RLUs in 10 min after lysing cells with 1% Triton X-100.

### 4.5. Multielemental Analysis of Yeast Cells

Metal accumulation by cells was done as described [74], with slight modifications. Exponentially YPD-growing cells were washed and suspended in MMe liquid medium to OD_600_ = 0.5 in the absence or presence of oleandrin (100 ng/mL). The cells were incubated with shaking (200 rpm) for 16 h at 30 °C before they were harvested and washed three times with 10 mM 2-(*N*-morpholino)ethanesulfonic acid (MES)-Tris buffer, pH 6.0. Cells were finally suspended in deionized water (10^8^ cells/mL) and used for both metal and cell protein assays. Metal detection was done using an instrument with a single collector, quadrupole inductively coupled plasma with mass spectrometry (ICP-MS) with axial field technology for trace elements, rare earth elements and isotopic analyses. Metal analyses were performed after digestion of cells with 65% ultrapure HNO_3_ (Merck). Standard solutions were prepared by diluting a 10 µg/mL multielement solution (Multielement ICP Calibration Standard 3, matrix 5%HNO_3_, Perkin Elmer Pure Plus, Shelton, CT, USA). The metal cellular content was normalized to total cellular proteins, which were assayed spectrophotometrically [75].

### 4.6. Statistics

All experiments were repeated, independently, in three biological replicates at least. For each individual experiment, values were expressed as the mean ± standard error of the mean (SEM). For aequorin luminescence determinations, traces represent the mean (±SEM) from three independent transformants. The numerical data were examined by Student *t* test or by analysis of variance with multiple comparisons (ANOVA) using the statistical software Prism version 6.05 for Windows (GraphPad Software, La Jolla, CA, USA). The differences were considered to be significant when *p* < 0.05. One sample *t* test was used for the statistical analysis of each strain/condition compared with a strain/condition considered as reference. Asterisks indicate the level of significance: * *p* < 0.05, ** *p* < 0.01 and *** *p* < 0.001.

## 5. Conclusions

Oleandrin toxicity against eukaryotic cells was investigated using the model microorganism *S. cerevisiae*. We found that exposing yeast cells to oleandrin resulted in Ca^2+^ influx into the cytosol and that defects in restoring the normal level of cytosolic Ca^2+^ (e.g., by pumping excess cytosolic Ca^2+^ to the vacuole) augmented the oleandrin toxicity. We also found that oleandrin exposure induced Mn^2+^ accumulation by the yeast cells via the plasma membrane Smf1 and that mutants with defects in Mn^2+^ homeostasis may become oleandrin-hypersensitive. Our data suggest that combining oleandrin with agents which alter Ca^2+^ or Mn^2+^ homeostasis may be a way of scope-tuning oleandrin toxicity.

## Figures and Tables

**Table 1:** Effect of oleandrin on metal content of yeast cells. Exponentially growing wild type cells were shifted to MMe (OD_600_ = 0.5) in the presence of oleandrin (100 ng/mL). Cells were grown with agitation for 16 h before being harvested for multi-elemental analysis, as described in the *Materials and Methods* section. Each determination was done in triplicate on approximately 10^8^ cells from three biological replicates. Results are given as mean ± standard deviation.

Metal Detected	Cellular Metal Content (nmoles/mg Total Cell Protein)	
	No Oleandrin	Oleandrin
Co^2+^	1.32 ± 0.24	1.44 ± 0.31
Cu^2+^	5.84 ± 0.42	5.52 ± 0.82
Fe^3+^	52.82 ± 3.24	54.33 ± 3.84
Mn^2+^	4.25 ± 0.62	9.92 ± 1.82 *
Ni^2+^	0.24 ± 0.12	0.22 ± 0.21
Zn^2+^	12.42 ± 1.14	11.88 ± 1.43
Li^+^	1.14 ± 0.32	1.21 ± 0.22

* Significantly different from control (no oleandrin) as of the one-sample *t* test, *p* < 0.05.

## Supplementary Materials

The following are available online, Table S1: Effect of oleandrin on the growth of various single-gene knockout mutants.

## References

[1] Dey P. (2020). The pharmaco-toxicological conundrum of oleander: Potential role of gutmicrobiome. Biomed. Pharmacother..

[2] Langford S.D., Boor P.J. (1996). Oleander toxicity: An examination of human and animal toxic exposures. Toxicology.

[3] Karthik G., Iyadurai R., Ralph R., Prakash V., Abhilash K.P.P., Sathyendra S., Abraham O.C., Truman C., Reginald A. (2020). Acute oleander poisoning: A study of clinical profile from a tertiary care center in South India. J. Fam. Med. Prim. Care.

[4] Botelho A.F.M., Santos-Miranda A., Joca H.C., Mattoso C.R.S., de Oliveira M.S., Pierezan F., Cruz J.S., Soto-Blanco B., Melo M.M. (2017). Hydroalcoholic extract from *Nerium oleander* L. (*Apocynaceae*) elicits arrhythmogenic activity. J. Ethnopharmacol..

[5] Rashan L.J., Franke K., Khine M.M., Kelter G., Fiebig H.H., Neumann J., Wessjohann L.A. (2011). Characterization of the anticancer properties of monoglycosidic cardenolides isolated from *Nerium oleander* and *Streptocaulon tomentosum*. J. Ethnopharmacol..

[6] Cao Y.L., Zhang M.H., Lu Y.F., Li C.Y., Tang J.S., Jiang M.M. (2018). Cardenolides from the leaves of *Nerium oleander*. Fitoterapia.

[7] Botelho A.F.M., Pierezan F., Soto-Blanco B., Melo M.M. (2019). A review of cardiac glycosides: Structure, toxicokinetics, clinical signs, diagnosis and antineoplastic potential. Toxicon.

[8] Hutchison T., Yapindi L., Malu A., Newman R.A., Sastry K.J., Harrod R. (2019). The botanical glycoside oleandrin inhibits human T-cell leukemia virus type-1 infectivity and Env-dependent virological synapse formation. J. Antivir. Antiretrovir..

[9] https://pubchem.ncbi.nlm.nih.gov/compound/11541511.

[10] Lin Y., Ho D.H., Newman R.A. (2010). Human tumor cell sensitivity to oleandrin is dependent on relative expression of Na^+^,K^+^-ATPase subunits. J. Exp. Ther. Oncol..

[11] Botelho A.F.M., Miranda A.L.S., Freitas T.G., Milani P.F., Barreto T., Cruz J.S., Melo M.M. (2020). Comparative cardiotoxicity of low doses of digoxin, ouabain, and oleandrin. Cardiovasc. Toxicol..

[12] Bao Z., Tian B., Wang X., Feng H., Liang Y., Chen Z., Li W., Shen H., Ying S. (2016). Oleandrin induces DNA damage responses in cancer cells by suppressing the expression of Rad51. Oncotarget.

[13] Pan L., Zhang Y., Zhao W., Zhou X., Wang C., Deng F. (2017). The cardiac glycoside oleandrin induces apoptosis in human colon cancer cells via the mitochondrial pathway. Cancer Chemother. Pharmacol..

[14] Ko Y.S., Rugira T., Jin H., Park S.W., Kim H.J. (2018). Oleandrin and its derivative odoroside a, both cardiac glycosides, exhibit anticancer effects by inhibiting invasion via suppressing the STAT-3 signaling pathway. Int. J. Mol. Sci..

[15] Yong L., Ma Y., Liang C., He G., Zhao Z., Yang C., Hai B., Pan X., Liu Z., Liu X. (2019). Oleandrin sensitizes human osteosarcoma cells to cisplatin by preventing degradation of the copper transporter 1. Phytother. Res..

[16] Li X.X., Wang D.Q., Sui C.G., Meng F.D., Sun S.L., Zheng J., Jiang Y.H. (2020). Oleandrin induces apoptosis via activating endoplasmic reticulum stress in breast cancer cells. Biomed. Pharmacother..

[17] Singh S., Shenoy S., Nehete P.N., Yang P., Nehete B., Fontenot D., Yang G., Newman R.A., Sastry K.J. (2013). *Nerium oleander* derived cardiac glycoside oleandrin is a novel inhibitor of HIV infectivity. Fitoterapia.

[18] Yang C.W., Chang H.Y., Hsu H.Y., Lee Y.Z., Chang H.S., Chen I.S., Lee S.J. (2017). Identification of anti-viral activity of the cardenolides, Na(+)/K(+)-ATPase inhibitors, against porcine transmissible gastroenteritis virus. Toxicol. Appl. Pharmacol..

[19] Plante K.S., Plante J.A., Fernandez D., Mirchandani D., Bopp N., Aguilar P.V., Sastry K.J., Newman R.A., Weaver S.C. (2020). Prophylactic and therapeutic inhibition of in vitro SARS-CoV-2 replication by Oleandrin. bioRxiv.

[20] Kanwal N., Rasul A., Hussain G., Anwar H., Shah M.A., Sarfraz I., Riaz A., Batool R., Shahba M., Hussain A. (2020). Oleandrin: A bioactive phytochemical and potential cancer killer via multiple cellular signaling pathway. Food Chem. Toxicol..

[21] Newman R.A., Yang P., Hittelman W.N., Lu T., Ho D.H., Ni D., Chan D., Vijjeswarapu M., Cartwright C., Dixon S. (2006). Oleandrin-mediated oxidative stress in human melanoma cells. J. Exp. Ther. Oncol..

[22] Castrillo J.I., Oliver S. (2004). Yeast as a touchstone in post-genomic research: Strategies for integrative analysis in functional genomics. J. Biochem. Mol. Biol..

[23] Matuo R., Sousa F.G., Soares D.G., Bonatto D., Saffi J., Escargueil A.E., Larsen A.K., Henriques J.A. (2012). *Saccharomyces cerevisiae* as a model system to study the response to anticancer agents. Cancer Chemother. Pharmacol..

[24] Dos Santos S.C., Sá-Correia I. (2015). Yeast toxicogenomics: Lessons from a eukaryotic cell model and cell factory. Curr. Opin. Biotechnol..

[25] Manna S.K., Sah N.K., Newman A., Cisneros A., Aggarwal B.B. (2000). Oleandrin suppresses activation of nuclear transcription factor-kB, activator protein-1, and c-Jun *N*-terminal kinase. Cancer Res..

[26] Ariño J., Ramos J., Sychrova H. (2019). Monovalent cation transporters at the plasma membrane in yeasts. Yeast.

[27] Matsumoto T.K., Ellsmore A.J., Cessna S.G., Low P.S., Pardo J.M., Bressan R.A., Hasegawa P.M. (2002). An osmotically induced cytosolic Ca^2+^ transient activates calcineurin signaling to mediate ion homeostasis and salt tolerance of *Saccharomyces cerevisiae*. J. Biol. Chem..

[28] Viladevall L., Serrano R., Ruiz A., Domenech G., Giraldo J., Barceló A., Ariño J. (2004). Characterization of the calcium-mediated response to alkaline stress in *Saccharomyces cerevisiae*. J. Biol. Chem..

[29] Bootman M.D., Berridge M.J., Putney J.W., Roderick H.L. (2011). Calcium Signaling.

[30] Batiza A.F., Schulz T., Masson P.H. (1996). Yeast respond to hypotonic shock with a calcium pulse. J. Biol. Chem..

[31] Denis V., Cyert M.S. (2002). Internal Ca(2+) release in yeast is triggered by hypertonic shock and mediated by a TRP channel homologue. J. Cell Biol..

[32] Palmer C.P., Zhou X., Lin J., Loukin S.H., Kung C., Saimi Y. (2001). A TRP homolog in *Saccharomyces cerevisiae* forms an intracellular Ca(2+)-permeable channel in the yeast vacuolar membrane. Proc. Natl. Acad. Sci. USA.

[33] Cunningham K.W. (2011). Acidic calcium stores of *Saccharomyces cerevisiae*. Cell Calcium.

[34] Paidhungat M., Garrett S. (1997). A homolog of mammalian, voltage-gated calcium channels mediates yeast pheromone-stimulated Ca^2+^ uptake and exacerbates the cdc1(Ts) growth defect. Mol. Cell. Biol..

[35] Iida K., Teng J., Cho T., Yoshikawa-Kimura S., Iida H. (2017). Post-translational processing and membrane translocation of the yeast regulatory Mid1 subunit of the Cch1/VGCC/NALCN cation channel family. J. Biol. Chem..

[36] Cunningham K.W., Fink G.R. (1994). Calcineurin-dependent growth control in *Saccharomyces cerevisiae* mutants lacking *PMC1*, a homolog of plasma membrane Ca^2+^ ATPases. J. Cell Biol..

[37] Cunningham K.W., Fink G.R. (1996). Calcineurin inhibits VCX1-dependent H^+^/Ca^2+^ exchange and induces Ca^2+^ ATPases in *Saccharomyces cerevisiae*. Mol. Cell. Biol..

[38] Miseta A., Kellermayer R., Aiello D.P., Fu L., Bedwell D.M. (1999). The vacuolar Ca^2+^/H^+^ exchanger Vcx1p/Hum1p tightly controls cytosolic Ca^2+^ levels in *S*. cerevisiae. FEBS Lett..

[39] Sorin A., Rosas G., Rao R. (1997). PMR1, a Ca^2+^-ATPase in yeast Golgi, has properties distinct from sarco/endoplasmic reticulum and plasma membrane calcium pumps. J. Biol. Chem..

[40] Dürr G., Strayle J., Plemper R., Elbs S., Klee S.K., Catty P., Wolf D.H., Rudolph H.K. (1998). The medial-Golgi ion pump Pmr1 supplies the yeast secretory pathway with Ca^2+^ and Mn^2+^ required for glycosylation, sorting, and endoplasmic reticulum-associated protein degradation. Mol. Biol. Cell.

[41] Culotta V.C., Yang M., Hall M.D. (2005). Manganese transport and trafficking: Lessons learned from *Saccharomyces cerevisiae*. Eukaryot. Cell.

[42] Kellermayer R. (2005). Hailey-Hailey disease as an orthodisease of *PMR1* deficiency in *Saccharomyces cerevisiae*. FEBS Lett..

[43] Van Ho A., Ward D.M., Kaplan J. (2002). Transition metal transport in yeast. Annu. Rev. Microbiol..

[44] Dancis A., Haile D., Yuan D.S., Klausner R.D. (1994). The *Saccharomyces cerevisiae* copper transport protein (Ctr1p). Biochemical characterization, regulation by copper, and physiologic role in copper uptake. J. Biol. Chem..

[45] Eide D.J. (1998). The molecular biology of metal ion transport in *Saccharomyces cerevisiae*. Annu. Rev. Nutr..

[46] Hassett R., Dix D.R., Eide D.J., Kosman D.J. (2000). The Fe(II) permease Fet4p functions as a low affinity copper transporter and supports normal copper trafficking in *Saccharomyces cerevisiae*. Biochem. J..

[47] Supek F., Supekova L., Nelson H., Nelson N. (1996). A yeast manganese transporter related to the macrophage protein involved in conferring resistance to mycobacteria. Proc. Natl. Acad. Sci. USA.

[48] Jensen L.T., Ajua-Alemanji M., Culotta V.C. (2003). The *Saccharomyces cerevisiae* high affinity phosphate transporter encoded by *PHO84* also functions in manganese homeostasis. J. Biol. Chem..

[49] Ofiteru A.M., Ruta L.L., Rotaru C., Dumitru I., Ene C.D., Neagoe A., Farcasanu I.C. (2012). Overexpression of the *PHO84* gene causes heavy metal accumulation and induces Ire1p-dependent unfolded protein response in *Saccharomyces cerevisiae* cells. Appl. Microbiol. Biotechnol..

[50] Zhao H., Eide D. (1996). The yeast *ZRT1* gene encodes the zinc transporter protein of a high-affinity uptake system induced by zinc limitation. Proc. Natl. Acad. Sci. USA.

[51] Zhao H., Eide D. (1996). The *ZRT2* gene encodes the low affinity zinc transporter in *Saccharomyces cerevisiae*. J. Biol. Chem..

[52] https://www.yeastgenome.org/.

[53] Nakajima-Shimada J., Iida H., Tsuji F.I., Anraku Y. (1991). Monitoring of intracellular calcium in *Saccharomyces cerevisiae* with an apoaequorine cDNA expression system. Proc. Natl. Acad. Sci. USA.

[54] Tisi R., Baldassa S., Belotti F., Martegani E. (2002). Phospholipase C is required for glucose-induced calcium influx in budding yeast. FEBS Lett..

[55] Mandal D., Woolf T.B., Rao R. (2000). Manganese selectivity of Pmr1, the yeast secretory pathway ion pump, is defined by residue gln783 in transmembrane segment 6. Residue Asp778 is essential for cation transport. J. Biol. Chem..

[56] Lee J., Spector D., Godon C., Labarre J., Toledano M.B. (1999). A new antioxidant with alkyl hydroperoxide defense properties in yeast. J. Biol. Chem..

[57] Farcasanu I.C., Hirata D., Tsuchiya E., Mizuta K., Miyakawa T. (1999). Involvement of thioredoxin peroxidase type II (Ahp1p) of Saccharomyces cerevisiae in Mn^2+^ homeostasis. Biosci. Biotechnol. Biochem..

[58] Kanzaki M., Nagasawa M., Kojima I., Sato C., Naruse K., Sokabe M., Iida H. (1999). Molecular identification of a eukaryotic, stretch-activated nonselective cation channel. Science.

[59] Erikson K.M., Aschner M., Sigel A., Freisinger E., Sigel R.K.O., Carver P.L. (2019). Manganese: Its role in disease and health. Essential Metals in Medicine: Therapeutic Use and Toxicity of Metal Ions in the Clinic.

[60] Farcasanu I.C., Mizunuma M., Nishiyama F., Miyakawa T. (2005). Role of L-histidine in conferring tolerance to Ni^2+^ in *Sacchromyces cerevisiae* cells. Biosci. Biotechnol. Biochem..

[61] Oprea E., Ruta L.L., Nicolau I., Popa C.V., Neagoe A.D., Farcasanu I.C. (2014). *Vaccinium corymbosum* L. (blueberry) extracts exhibit protective action against cadmium toxicity in *Saccharomyces cerevisiae* cells. Food Chem..

[62] Lapinskas P.J., Cunningham K.W., Liu X.F., Fink G.R., Culotta V.C. (1995). Mutations in *PMR1* suppress oxidative damage in yeast cells lacking superoxide dismutase. Mol. Cell. Biol..

[63] Au C., Benedetto A., Aschner M. (2008). Manganese transport in eukaryotes: The role of DMT1. Neurotoxicology.

[64] Courville P., Chaloupka R., Cellier M.F. (2006). Recent progress in structure-function analyses of Nramp proton-dependent metal-ion transporters. Biochem. Cell Biol..

[65] Calhoun E.S., McGovern R.M., Janney C.A., Cerhan J.R., Iturria S.J., Smith D.I., Gostout B.S., Persing D.H. (2002). Host genetic polymorphism analysis in cervical cancer. Clin. Chem..

[66] Lenormand C., Couteau J., Nouhaud F.X., Maillet G., Bou J., Gobet F., Pfister C. (2016). Predictive value of NRAMP1 and HGPX1 gene polymorphism for maintenance BCG response in non-muscle-invasive bladder cancer. Anticancer Res..

[67] Hernroth B., Holm I., Gondikas A., Tassidis H. (2018). Manganese inhibits viability of prostate cancer cells. Anticancer Res..

[68] Doble P.A., Miklos G.L.G. (2018). Distributions of manganese in diverse human cancers provide insights into tumour radioresistance. Metallomics.

[69] Brachmann C.B., Davies A., Cost G.J., Caputo E., Li J., Hieter P., Boeke J.D. (1998). Designer deletion strains derived from *Saccharomyces cerevisiae* S288C: A useful set of strains and plasmids for PCR-mediated gene disruption and other applications. Yeast.

[70] Sherman F. (2002). Getting started with yeast. Methods Enzymol..

[71] Cohen R., Engelberg D. (2007). Commonly used *Saccharomyces cerevisiae* strains (e.g., BY4741, W303) are growth sensitive on synthetic complete medium due to poor leucine uptake. FEMS Microbiol. Lett..

[72] Dohmen R.J., Strasser A.W.M., Honer C.B., Hollenberg C.P. (1991). An efficient transformation procedure enabling long-term storage of competent cells of various yeast genera. Yeast.

[73] Tisi R., Martegani E., Brandão R.L. (2015). Monitoring yeast intracellular Ca^2+^ levels using an in vivo bioluminescence assay. Cold Spring Harb. Protoc..

[74] Ruta L.L., Kissen R., Nicolau I., Neagoe A.D., Petrescu A.J., Bones A.M., Farcasanu I.C. (2017). Heavy metal accumulation by *Saccharomyces cerevisiae* cells armed with metal binding hexapeptides targeted to the inner face of the plasma membrane. Appl. Microbiol. Biotechnol..

[75] Bradford M.M. (1976). A rapid and sensitive method for the quantitation of microgram quantities of protein utilizing the principle of protein-dye binding. Anal. Biochem..

