# Supplementary material for: Cytotoxicity of Oleandrin Is Mediated by Calcium Influx and by Increased Manganese Uptake in Saccharomyces cerevisiae Cells
Source: Molecules. 2020 Sep 17;25(18):4259. doi: 10.3390/molecules25184259 (PMC7570853; doi:10.3390/molecules25184259)
Supplement: Supplementary file 1 [file molecules-25-04259-s001.pdf]

Supplementary Materials for

Cytotoxicity of Oleandrin Is Mediated by Calcium Influx and by Increased Manganese Uptake in *Saccharomyces cerevisiae* Cells

Table S1. Effect of oleandrin on the growth of various single-gene knockout mutants of *Saccharomyces cerevisiae*. Strains were inoculated (initial cell density 5 × 10^5 cells/mL) in SD media containing 100 ng/mL oleandrin (final concentration). Growth was assessed spectrophotometrically (OD_{660}) after 16 hours of incubation with shaking (200 rpm) at 30 °C and expressed (%) as the ration between cell density in the presence of oleandrin and cell density in the absence of oleandrin.

Strain	Gene Deleted	Gene Function	Growth in the Presence of Oleandrin (%)
Wild type [1]	None	Control strain	49.6 ± 2.8
		Genes involved in monovalent cation transport	
ena1Δ	*ENA1*	P-type ATPase sodium pump; involved in Na^+ and Li^+ efflux.	51.2 ± 1.8
nha1Δ	*NHA1*	Na^+/H^+ antiporter; involved in sodium and potassium efflux through the plasma membrane	54.6 ± 2.5
tok1Δ	*TOK1*	Outward-rectifier potassium channel of the plasma membrane	47.2 ± 3.4
trk1Δ	*TRK1*	High affinity K^+ transporter	53.1 ± 2.4
trk2Δ	*TRK2*	Trk1 paralog; K^+ transporter	51.6 ± 1.9
		Genes involved in calcium homeostasis	
cch1Δ	*CCH1*	Voltage-gated high-affinity Ca^{2+} channel involved in calcium influx in response to some stresses	78.8 ± 6.9 *
mid1Δ	*MID1*	Stretch-activated Ca^{2+}-permeable cation channel; interacts with Cch1	89.6 ± 7.9 **
pmc1Δ	*PMC1*	Vacuolar Ca^{2+}-ATPase involved in depleting cytosol of Ca^{2+} ions.	21.6 ± 1.7 **
vcx1Δ	*VCX1*	Vacuolar membrane antiporter with Ca^{2+}/H^+ and K^+/H^+ exchange activity	43.6 ± 2.9
yvc1Δ	*YVC1*	Vacuolar cation channel; mediates release of Ca^{2+} from the vacuole	57.6 ± 4.9
pmr1Δ	*PMR1*	High affinity Ca2+/Mn2+ P-type ATPase; required for Ca^{2+} and Mn^{2+} transport into Golgi	16.8 ± 2.1
		Genes Involved in Heavy Metal Transport	

ctr1Δ	*CTR1*	High-affinity copper transporter of plasma membrane	48.6 ± 3.4
ftr1Δ	*FTR1*	High-affinity iron transporter of the plasma membrane	49.2 ± 4.3
fet4Δ	*FET41*	Low-affinity iron transporter of the plasma membrane	54.6 ± 4.5
smf1Δ	*SMF1*	Divalent metal ion transporter	91.6 ± 4.9 ***
pho84Δ	*PHO84*	High-affinity inorganic phosphate (Pi) transporter and low-affinity divalent metal ion transporter	54.3 ± 3.7
ztr1Δ	*ZTR1*	High-affinity Zn^{2+} transporter of the plasma membrane	53.6 ± 3.9
ztr2Δ	*ZTR2*	Low-affinity Zn^{2+} transporter of the plasma membrane	54.5 ± 4.2
		Genes involved response to oxidative stress	
yap1Δ	*YAP1*	Transcription factor; required for oxidative stress tolerance	41.6 ± 5.2
skn7Δ	*SKN7*	Transcription factor required for optimal induction of heat-shock genes in response to oxidative stress	43.8 ± 6.1
msn2Δ	*MSN2*	Stress-responsive transcriptional activator	50.6 ± 6.8
sod1Δ	*SOD1*	Cu,Zn-Superoxide dismutase	48.6 ± 4.2
sod2Δ	*SOD2*	Mn-Superoxide dismutase	47.3 ± 4.6
ahp1Δ	*AHP1*	Thioredoxin peroxidase with alkyl hydroperoxide reductase activity	28.3 ± 3.2 **
ccp1Δ	*CCP1*	Citocrome c peroxidase	48.6 ± 5.1
cta1Δ	*CTA1*	Catalase A	47.3 ± 4.8
ctt1Δ	*CTT1*	Calalase T	48.6 ± 5.2
gpx1Δ	*GPX1*	Phospholipid hydroperoxide glutathione peroxidase	513 ± 4.9
gpx2Δ	*GPX2*	Phospholipid hydroperoxide glutathione peroxidase	43.4 ± 4.1
hyr1Δ	*HYR1*	Thiol peroxidase that functions as a hydroperoxide receptor to sense intracellular hydroperoxide levels and transduce a redox signal to the Yap1p transcription factor	49.3 ± 5.7

prx1Δ	*PRX1*	Mitochondrial peroxiredoxin with thioredoxin peroxidase activity	44.6 ± 6.3
trr1Δ	*TRR1*	Cytoplasmic thioredoxin reductase	54.6 ± 7.5
tsa1Δ	*TSA1*	Thioredoxin peroxidase; acts as both ribosome-associated and free cytoplasmic antioxidant	48.3 ± 4.9
tsa2Δ	*TSA2*	Stress inducible cytoplasmic thioredoxin peroxidase	41.6 ± 6.2

[1] Wild type is strain BY4741 (*MAT***a**; *his3Δ1*; *leu2Δ0*; *met15Δ0*; *ura3Δ0*). The isogenic knock-out mutants had the phenotype BY4741 *yfg: kanMX4* except for a null mutation of one single gene deletion. All strains were purchased from EUROSCARF (www.euroscarf.de).
